# Structural Model for the Effects of Environmental Elements on the Psychological Characteristics and Performance of the Employees of Manufacturing Systems

**DOI:** 10.3390/ijerph13010104

**Published:** 2016-01-05

**Authors:** Arturo Realyvásquez, Aidé Aracely Maldonado-Macías, Jorge García-Alcaraz, Guillermo Cortés-Robles, Julio Blanco-Fernández

**Affiliations:** 1 Department of Industrial and Manufacturing Engineering, Autonomous University of Ciudad Juarez, Del Charro Ave., 450 N., Ciudad Juárez, Chihuahua, 32310, México; amaldona@uacj.mx (A.A.M.-M.); jorge.garcia@uacj.mx (J.G.-A.); 2 Department of Mechanical Engineering, University of La Rioja, San José de Calasanz 31, Logroño, La Rioja 26004, Spain; julio.blanco@unirioja.es; 3 Technological Institute of Orizaba, Oriente 9, 852, Orizaba, Veracruz 94320, Mexico; gc_robles@hotmail.com

**Keywords:** macroergonomics, macroergonomic compatibility questionnaire, environment, personnel characteristics, structural equations modeling, manufacturing systems

## Abstract

This paper analyzes the effects of environmental elements on the psychological characteristics and performance of employees in manufacturing systems using structural equation modeling. Increasing the comprehension of these effects may help optimize manufacturing systems regarding their employees’ psychological characteristics and performance from a macroergonomic perspective. As the method, a new macroergonomic compatibility questionnaire (MCQ) was developed and statistically validated, and 158 respondents at four manufacture companies were considered. Noise, lighting and temperature, humidity and air quality (THAQ) were used as independent variables and psychological characteristics and employees’ performance as dependent variables. To propose and test the hypothetical causal model of significant relationships among the variables, a data analysis was deployed. Results found that the macroergonomic compatibility of environmental elements presents significant direct effects on employees’ psychological characteristics and either direct or indirect effects on the employees’ performance. THAQ had the highest direct and total effects on psychological characteristics. Regarding the direct and total effects on employees’ performance, the psychological characteristics presented the highest effects, followed by THAQ conditions. These results may help measure and optimize manufacturing systems’ performance by enhancing their macroergonomic compatibility and quality of life at work of the employees.

## 1. Introduction

Analysis of macroergonomic factors, such as the person, organization, technology, tasks and environment, is relevant for guiding manufacturing systems toward competitiveness in an increasingly saturated market place. Ergonomic compatibility considers the concepts of human-system and human-artifact compatibility, which were introduced by [1,2]. These concepts emerged from the need for having a more comprehensive treatment of compatibility in the human factor discipline [1,3]. Several authors, such as [1,2,3,4,5,6], have addressed ergonomic compatibility either at the micro or the macro level. The authors explain that macroergonomic compatibility exists when a work system supports the appropriate interaction between the personnel subsystem and the technological subsystem. This interaction includes the work system’s relationship with external environment characteristics. Nevertheless, there is not yet an available model for the validation and evaluation of the macroergonomic compatibility construct in manufacturing systems.

Macroergonomic compatibility can produce benefits for manufacturing systems, from process optimization and reduction of occupational risks to the notable improvement of life quality at the workplace. These benefits can be achieved by applying macroergonomic practices to work system design [4,7]. The correct design of a work system will determine its effectiveness [8,9]. A work system is defined as a system in which human participants and/or machines perform different tasks using information, technology and other resources to produce products and services for internal or external customers [10].

This paper analyzes the effects of environmental elements on the psychological characteristics and performance of employees in manufacturing systems using structural equation modeling (SEM). Environmental elements include the noise level, lighting and the so-called temperature, humidity and air quality (THAQ) conditions, which are the temperature, humidity and air quality. According to [11], the environment factor primarily focuses on the physical environment, including lighting, noise level and THAQ conditions.

In this paper, a new macroergonomic compatibility questionnaire (MCQ) for manufacturing systems is designed and statistically validated, regarding relevant models, such as the systems engineering initiative for patient safety (SEIPS) model developed by [12,13]. The factors and their corresponding elements used in this paper are: (1) the person factor, with the element of psychological characteristics; (2) the organization, with the elements of employees’ performance; and (3) the environment, which includes the elements of noise, lighting and THAQ conditions.

This research does not merely focus on analyzing the effects of environmental elements on the psychological characteristics of employees in manufacturing systems; it also analyzes the impact of these psychological characteristics on human performance that can eventually diminish organizational performance over the long term.

This study also offers an integrated approach to understanding the macroergonomic compatibility of environmental elements and their direct and indirect effects on two fundamental axes: psychological characteristics and employees’ performance in manufacturing systems. As far as the psychological aspects are concerned, this study considers the psychological characteristics of employees as independent variables that include task design and allocation, the psychological support available and the attention given to employees to analyze and detect the causes of their psychological distress, as well as the employees’ satisfaction with the task design. On the other hand, in terms of employees’ performance, this paper intends to find out whether employees are encouraged to give their maximum effort through different means, such as task-incentive programs.

As a result, this paper proposes and tests a hypothetical casual model that searches for the relevant relationships among the constructs by using a structural equation modeling approach. Results derived from the model can offer relevant and new information to develop strategies for manufacturing systems to increase their competitiveness. Moreover, determining the direct and indirect effects of environmental factors, as well as identifying those with a positive impact on the manufacturing systems’ performance could be an effective business strategy for manufacturing systems’ design, since all of these effects could become aspects of competitiveness and favor economical results. The section of this paper named “Industrial Implications” offers several recommendations for the manufacturing systems participating in this study; however, some companies interested in this article may demonstrate resistance to considering macroergonomic practices, since manufacturing systems usually look for short-term benefits.

## 2. Literature Review and Hypothetical Causal Model

By using keywords, such as macroergonomics, work systems, environment, noise, lighting, *etc.*, the literature was reviewed in several databases (Science Direct, Google Scholar, Ebsco Host and Elsevier) to explore the presence of the environment elements mentioned above for an ergonomically-compatible work systems’ design. According to this literature, environmental elements have been neglected in the design of compatible work systems. Furthermore, the literature revealed that environmental elements have significant short-term effects on human psychology and performance and long-term effects on a company’s competitiveness.

Among the environmental elements, lighting is the most frequently mentioned in the literature. It is stated that workplace lighting is an essential element to determine the employees’ errors, accidents, absenteeism, wellbeing and productivity as an overall effect [14,15,16,17]. Noise level was the second environmental element most frequently cited, followed by THAQ conditions. It is argued that noise causes negative effects on human health and task performance [18]. Meanwhile, for THAQ conditions, temperatures above 25 °C can result in decreasing employees’ performance [19]. Humidity also has an effect on human performance [20], and air quality impacts on health and the employees’ satisfaction with their workplace [21,22,23,24,25].

Additionally, psychological characteristics were found to be one of the personnel characteristics less frequently found in literature, which gave them little attention for the design of work systems and the achievement of goals in companies. Nevertheless, some psychological characteristics, such as work stress, have been associated with psychological reactions, such as depressive symptoms, insomnia and job dissatisfaction [26].

The new MCQ was used to get information about macroergonomic practices in manufacturing systems. This information served to measure the effects of the macroergonomic compatibility of elements on employees’ performance. In the following subsections, a definition of macroergonomic compatibility is given and, also, the literature about the impact of environmental elements on employees’ psychological characteristics and performance. These studies served as the basis to propose and validate the hypotheses of the hypothetical model.

### 2.1. Macroergonomic Compatibility Construct

Macroergonomics is the study of work systems that focuses on the achievement of a fully-harmonized work system at both the macroergonomic and microergonomic levels [8,27,28,29]. A keyword to define ergonomics is “compatibility”. Traditionally, this concept has been defined in different ways by relating it to the term “fitting” [5,6]. According to [5,30], the American Heritage Dictionary of the English Language defines “compatible” as: (1) capable of living or performing in harmonious, agreeable or congenial combination with another or others; and as being (2) capable of having orderly, efficient integration and operation with other elements in a system. Therefore, it can be said that “compatibility” is the capability of living or performing in harmonious, agreeable and efficient operation with other elements of the same system. However, this concept is still open to intuitive interpretation from each reader in most of the literature about ergonomics. Therefore, to avoid different interpretations of the concept, Karwowski [5] proposed the Symvatology, a corroborative science to ergonomics, with the final goal of developing a universal measure of compatibility for design, testing and evaluation of ergonomic systems [3,5,6].

According to [6], compatibility at a microergonomic level refers to the extent to which elements of a system (tools, devices, users and physical spaces) can interact without affecting themselves or the system’s purpose negatively. The authors also stated that macroergonomic compatibility or compatibility in multiplicity can be obtained when a work system (structure and related processes) supports an appropriate interaction with the personnel subsystem and the technological subsystem, including the work system’s relation with external environment characteristics.

The following subsections address the environmental aspects analyzed in this research, as well the effects that they cause. The review also supports the hypothetical casual model.

### 2.2. Noise

Noise is defined as “unwanted sound”, and it is perceived as an environmental stressor and nuisance [31]. According to research, noise has negative effects on human health and task performance. These effects are classified into auditory and non-auditory effects [18]. Chao *et al.* [18], as well as Konings, van Laer and van Camp [32] found that while an auditory effect is hearing loss, non-auditory effects involve a fast heartbeat, high blood pressure [33], muscle contraction leading to fatigue and reduction of light sensitivity. Other authors, such as Stansfeld and Matheson [31], found that noise may cause hypertension, cardiovascular diseases, psychological symptoms (e.g., aggression and mental disorders) and loss of memory. The parameters that determine the severity of these effects are: (1) noise level; (2) exposure time; (3) characteristics of the noise frequency; and (4) different individual characteristics [18,34].

However, not all noise effects are directly related to health problems. In fact, it is said that noise can equally affect the employees’ performance. For instance, Stansfeld and Matheson [31] found that noise can interfere with the task performance. Furthermore, Sloof and Van Praag [35] performed an experiment with two groups of employees to determine the effects of high levels of noise. The first group worked in an environment with a stable noise level and the second one in an environment with volatile noise levels. Results showed that subjects working in the volatile environment had to make more efforts to conclude their tasks than those people who worked in stable noise levels.

Saeki, Fujii, Yamaguchi and Harima [36] and Dockrell and Shield [37] also performed different experiments to test the effect of noise levels on human performance, and in all of their cases, the authors concluded that participants had better performance when noise was at its lowest level. Other studies suggested that noise influences employees’ attitudes, behaviors, feelings of satisfaction and working performance [38,39,40,41] and that it can also be an environmental stressor related to job satisfaction [41,42]. Finally, Vischer [20] stated that noise is a primary source of discomfort that reduces productivity.

The information and evidence mentioned above proving that noise has negative effects on human health and performance help support the following hypotheses (H_1_ and H_2_) from a macroergonomic approach in manufacturing systems:

H_1_: Macroergonomic compatibility of noise in manufacturing systems has a direct and positive effect on employees’ psychological characteristics.
H_2_: Macroergonomic compatibility of noise in manufacturing systems has a direct and positive effect on employees’ performance.

### 2.3. Lighting

Since the end of the 1990s, quality lighting has balanced the needs of humans, as well as the economic and environmental aspects of life [43]. In the workplace, lighting is an environmental element that can impact to a great extent people’s health and performance [15]. In fact, according to certain authors, workplace lighting is a key element to determine the employees’ errors, accidents, absenteeism, well-being and productivity as an overall effect [14,15,16,17]. For instance, appropriate lighting for screen-based work helps ensure employees functional comfort at work [20]. Furthermore, Bellia, Bisegna and Spada [43] and Vischer [20] pointed out that, in addition to noise, inadequate or insufficient lighting exposure may generate stress and influence individual task performance, which often results in negative effects on productivity. Similarly, other studies have shown that lighting has serious psychological consequences on people, such as mental fatigue, slow response time on tasks, negative changes in attitudes and behaviors and less satisfaction [41,44]. Thus, adequate lighting can help employees feel less sleepy, more energetic and happier [45]. For instance, van Bommel, van den Beld and van Ooyen [46] calculated the possible total boost of productivity as the result of improved lighting and found that an improvement in the illumination of work places increases productivity up to 80%.

The previous information can support the following Hypotheses H_3_ and H_4_ concerning the effects of lighting on people’s health and performance from a macroergonomic perspective applied to manufacturing systems:

H_3_: Macroergonomic compatibility of lighting in manufacturing systems has a direct and positive effect on employees’ psychological characteristics.
H_4_: Macroergonomic compatibility of lighting has a direct and positive effect on employees’ performance.

### 2.4. Temperature, Humidity and Air Quality Conditions

Temperature, humidity and air quality (THAQ) conditions involve temperature, humidity and air quality. Temperature is defined as a physical magnitude that expresses the extent or level of heat or coolness of bodies or the environment [47]. Humidity, on another hand, is classified into two categories: absolute humidity (AH) and relative humidity (RH). AH refers to the absolute amount of water in the air, and RH is defined as the relative proportion of water in the air in comparison to the maximum water vapor [48]. This paper considers humidity as a single factor, but by considering the two aspects. Meanwhile, air quality refers to the level of air pollutants, which are controlled by the air quality standards [49].

All of these variables have been analyzed in previous research to study the effects they cause on different aspects of a working center, including the employees. Furthermore, several works have addressed the relationship between air temperature and human performance; nevertheless, most of this research focuses on non-manufacturing contexts. For instance, Niemelä *et al.* [19] reported that employees’ performance tended to decrease in call centers when the temperature was above 25 °C. Similarly, Pepler and Warner [50] found an inverse U-shape relationship between the time taken to complete a task and the temperature in the work place. Furthermore, Wyon [51] discovered that productivity loss due to inappropriate levels of temperature was strongly related to the nature of the task that employees had to perform.

Several studies supported the hypothesis that there is a range of temperatures that does not affect task performance [52,53]. Lorsch [54] stated that there was a critical temperature zone (between 32.2 °C and 35 °C) above which the accuracy of the performance of mental tasks declined. Similarly, Seppanen, Fisk and Lei [55] and Cui *et al.* [56] found that the highest productivity of office employees was at 22 °C, and that at a higher or lower temperature, productivity decreased. Similarly, some other studies found that temperature has an effect on learning processes and motivation, which, in turn, have an impact on performance [56,57,58].

Moreover, uncomfortable temperature influences not only employees’ performance, but also these employees’ health. According to Vischer [20], psychological aspects are related to workspace elements and thereby to organizational productivity. Some studies also revealed that temperature has an effect on employees’ attitudes, behaviors and performance [38,39,40,41], and what is more, most of the psychological effects of temperature are related to aggressive behaviors [59]. For example, Baron and Bell [60] performed several experiments with undergraduate students in order to find the effects of temperature on their behavior. Findings showed that high ambient temperature produced aggressive behaviors. On the other hand, Vrij, van der Steen and Koppelaar [61] analyzed the impact of temperature on police officers’ behavior and also found that increased temperature resulted in aggressive behavior.

As far as humidity is concerned, it is said that its high level affects individual task performance [20]. Tsutsumi *et al.* [62] conducted subjective experiments to evaluate the effects of humidity on human performance under transient conditions from a hot and humid environment to a thermally neutral condition. The results indicated that subjective performance was at the same level under all conditions. However, subjects reported feeling more tired at 70% RH after humidity step change. Similarly, Shi, Zhu and Zheng [63] pointed out that humidity had effects on physiological parameters, such as heart rate, body temperature, blood pressure and sweat, which would impact performance.

Merely a few studies have addressed the relationship between air quality and human performance in manufacturing systems. However, several authors that analyzed this relation in other office works found that air quality had a significant impact on productivity [64,65]. For instance, Huizenga, Abbaszadeh, Zagreus and Arens [64] performed a survey to ask building employees if air quality in their workspace enhanced or interfered with their ability to accomplish their tasks. The results obtained demonstrated that air quality did have a significant influence on task performance. Wargocki *et al.* [66] also agreed that good air quality had a positive impact on office employees’ performance. Finally, other studies have shown that air quality has an impact on the employees’ satisfaction [21,22,23,24], which, in turn, is positively correlated with the self-estimated productivity of employees [24].

Polluted air is also responsible for a great number of diseases and problems, such as cancer, anemia, impaired coordination, gait abnormalities, driving inability, lack of attention and concentration and poor cognitive performance. Moreover, high exposure to polluted air can cause psychological disturbances lasting for weeks or months [25]. The probability for a person to suffer from the presence of a contaminant depends on several aspects, such as the individual’s sensitivity to that contaminant, his or her psychological and physical health, the contaminant’s level of concentration in the air and the duration and frequency of the exposure [67].

Similarly, air pollution affects the stability and wellbeing of a region. For instance, in the case of building workers, sick building syndrome symptoms can have more serious consequences for public health and the economy than other major diseases due to widespread absenteeism and lowered productivity amongst the affected employees [68]. Furthermore, it has been estimated that the annual cost of headaches amongst the employees of the United States Environmental Protection Agency is as high as 2 million American dollars (USD) [68].

Previous information can support Hypotheses H_5_, H_6_, and H_7_ explaining the relationships between THAQ conditions and the employees’ performance from a macroergonomic perspective in manufacturing systems:

H_5_: Macroergonomic compatibility of THAQ conditions in manufacturing systems has a direct and positive effect on employees’ psychological characteristics.
H_6_: Macroergonomic compatibility of THAQ conditions has a direct and positive effect on employees’ performance.
H_7_: Macroergonomic compatibility of psychological characteristics has a direct and positive effect on employees’ performance.

Based on all of the literature reviewed, it can be theorized that macroergonomic compatibility between these environmental elements and the psychological characteristics of employees contributes to the health of employees, and it also produces short-term benefits for the company aiming to optimize manufacturing systems in the long term.

**Figure 1:**
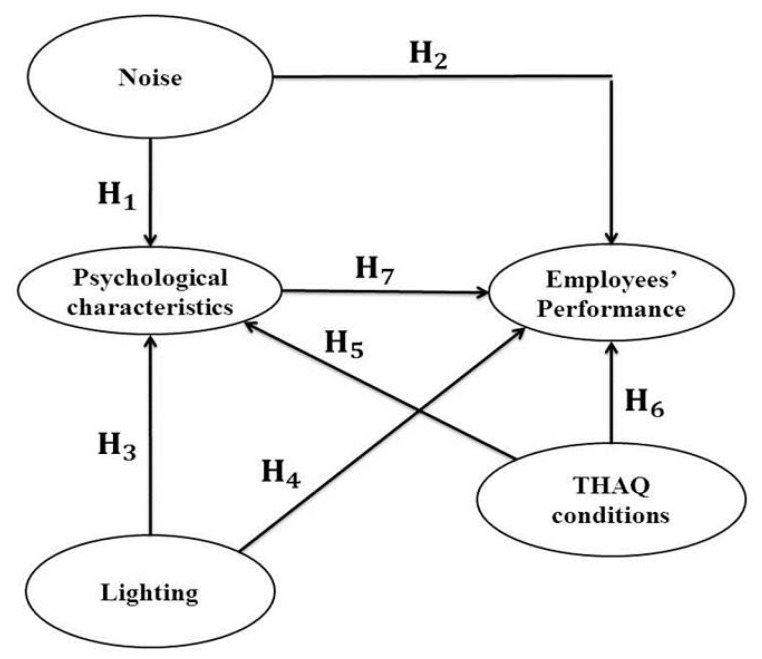
The proposed hypothetical causal model. THAQ, temperature, humidity and air quality.

All of the hypotheses mentioned above are shown in the hypothetical causal model depicted in Figure 1.

The environmental elements are independent latent variables; the psychological characteristics corresponds to an intermediate latent variable; and the employees’ performance is the final dependent latent variable.

## 3. Methodology

The methodology used in this study comprises the following stages: (1) macroergonomic compatibility questionnaire (MCQ) development; (2) application of the questionnaire; (3) statistical analysis; and (4) results interpretation.

### 3.1. Macroergonomic Compatibility Questionnaire Development

The literature presents several macroergonomic methods (e.g., interview, focus group, participatory ergonomics, laboratory experiment, macroergonomic analysis of structure (MAS), macroergonomic analysis and design (MEAD), *etc.*), which help identify some work systems’ design problems [69]. However, there is no method that pursues a macroergonomic compatibility measurement or index and can relate macroergonomic variables of compatibility with manufacturing systems’ performance for the clients, process and growth of companies. For example, the macroergonomic method of the macroergonomic organizational questionnaire survey (MOQS) [70] rather than being a questionnaire is a methodology to develop macroergonomic questionnaires; MEAD is a method that assesses work systems analyzing environment and organizational subsystems based on the variances of what is professed and what is practiced at the task and process level [69], but it does not provide a tool to assess the macroergonomic compatibility of work systems; MAS helps identify work systems’ discrepancies and correct them for improving work systems’ performance. Therefore, current macroergonomic methods are focused on improving work systems’ design and not on assessing macroergonomic compatibility.

Based on these backgrounds, a new macroergonomic compatibility questionnaire (MCQ) is developed. The MCQ has four main sections: (1) demographic data; (2) the employees’ perception regarding the implementation of specific macroergonomic practices in their companies; (3) the frequency of the application of macroergonomic practices in the company; and (4) the extent to which the company enjoys specific benefits.

Since the aim of the MCQ is to measure the macroergonomic compatibility of manufacturing systems, a literature review is conducted in order to determine the macroergonomic environmental elements (independent latent variables) that impact the macroergonomic compatibility of manufacturing systems, as well as the benefits that receive this impact (dependent latent variables). For this, the systems engineering initiative for patient safety (SEIPS) model of [12,13] is used as a reference model, since it includes a detailed description of the environmental elements that are significant to improve work systems from a macroergonomic perspective. Moreover, we use the principles and benefits of lean manufacturing set by [71] to determine the latent dependent variables. We use them because they mainly focus on defects, productivity and a variety of products.

The MCQ includes all of the elements of the factor “environment”: (1) noise; (2) lighting; and (3) THAQ conditions. Afterward, certain questions about these elements are derived from the literature studied, and the constructs are defined. Items are formulated from an extensive literature review and adapted for a macroergonomic perspective in manufacturing systems. Table 1 shows the references for the items’ formulation.

**Table 1:** References for the macroergonomic compatibility questionnaire (MCQ) items.

Item	Reference
*Noise*	
1.1a Tasks are performed in environments with comfortable noise levels	[26,72,73,74]
1.1b Employees are isolated from machines that emit a high noise level	[26,72,73,74]
1.1c Periodical noise level evaluations are performed	
*Lighting*	
1.2a Employees perform tasks at a safe and efficient lighting level	[26]
1.2b Periodical noise level evaluations are performed	
*THAQ Conditions*	
1.3a Employees perform tasks in comfortable THAQ conditions	[26,72,73,74]
1.3b Employees are isolated from machines that emit a high temperature	[26,72,73,74]
1.3c Employees perform tasks in an environment with clean air (e.g., without pollutants)	[26,72,73,74]
1.3d Periodical evaluations of THAQ conditions are performed	[72]
1.3e Employees use personal protection equipment against noise, uncomfortable THAQ conditions and air pollutants when it is required	[26,73,74]
*Psychological Characteristics*	
1.4a Employees’ psychological characteristics are considered for task assignment	[26]
1.4b Employees who manifest psychological diseases (e.g., mental stress, depression, *etc.*) receive medical attention	[26]
1.4c The causes of the psychological diseases of employees are analyzed	[26]
1.4d The tasks are designed in order to avoid psychological diseases (e.g., mental stress, depression, *etc.*) in employees	[26,75]
1.4e The tasks are designed in order to generate satisfaction in the employees	[26]
*Employees’ Performance*	
1.5a Employees are encouraged to make their best effort	[76]
1.5b The employees’ salary is commeasured according to the tasks they perform	[76]
1.5c All of the tasks are associated with incentives	[76]
1.5d Periodical evaluations of employees’ performance are made	

The search is conducted in the following databases: Science Direct, Google Scholar, PubMed/MEDLINE, Taylor & Francis, Wiley Online Library and SAGE. Once the MCQ is screened, judges handle its validation. This validation includes suggestions about the number of questions, reading comprehension issues and the readability aspects of the items. The original version of the MCQ is written in Spanish. However, an English version is available for employees of middle and senior management who only spoke English.

The MCQ has to be answered on a 5-point Likert scale; this scale is adopted since it has been used in recent and similar studies in [77,78,79]. Table 2 shows the scale used in the MCQ that answers the general question: “Does your company apply these macroergonomic practices?” and/or “Does your company enjoy of the following benefits?”

**Table 2:** The scale used to answer the MCQ.

1	2	3	4	5
Totally disagree	Disagree	Neither agree nor disagree	Agree	Totally agree

The first section of the MCQ includes questions about the general information of the company, its type of manufacturing system and the macroergonomic methods applied in it. The second section includes 92 items about ergonomic practices on the five factors (person, organization, tools and technology, tasks, environment) of the work systems. The third section includes 22 items to determine the rate at which companies apply macroergonomic practices on the elements of work systems. The fourth section includes 18 questions about the amount and type of benefits enjoyed by the companies when they apply macroergonomic practices. This section is formulated to ensure that companies that apply more macroergonomic practices also have more and better benefits. The last part of the MCQ contains the observations section so that responders can add their comments about the macroergonomic status of their manufacturing systems. Table 3, Table 4 and Table 5 show a sample of the different sections of the MCQ.

**Table 3:** Environmental aspects considered in the MCQ.

Environmental Aspects Assessment					
In Your Company:					
	Totally Disagree	Strongly Disagree	Neither Agree Nor Disagree	Strongly Agree	Totally Agree
1.1a Tasks are performed in environments with comfortable noise levels	1	2	3	4	5
1.1b Employees are isolated from machines that emit a high noise level	1	2	3	4	5
1.1c Periodical noise level evaluations are performed	1	2	3	4	5
1.2a Employees perform tasks at a safe and efficient lighting level	1	2	3	4	5
1.2b Periodical noise level evaluations are performed	1	2	3	4	5
1.3a Employees perform tasks in comfortable THAQ conditions	1	2	3	4	5
1.3b Employees are isolated from machines that emit a high temperature	1	2	3	4	5
1.3c Employees perform tasks in an environment with clean air (e.g., without pollutants)	1	2	3	4	5

**Table 4:** Section of the MCQ related to the frequency of application of macroergonomic practices.

Macroergonomic Practices on the Following Macroergonomic Elements Are Performed with a Frequency:					
	Very Low	Low	Medium	High	Very High
1.1 Noise	1	2	3	4	5
1.2 Lighting	1	2	3	4	5
1.3 Temperature, humidity and air quality	1	2	3	4	5
1.4 Employees’ psychological characteristics	1	2	3	4	5

**Table 5:** Benefits considered in the MCQ when macroergonomic practices are applied.

In Your Company:					
	Totally Disagree	Strongly Disagree	Neither Agree Nor Disagree	Strongly Agree	Totally Agree
1.a Needs and expectations from clients are considered	1	2	3	4	5
1.b Clients are satisfied with the products they receive	1	2	3	4	5
1.c Clients are loyal to the company	1	2	3	4	5
1.d The number of clients increases over the time	1	2	3	4	5
2.a The number of complaints by clients is very low	1	2	3	4	5
2.b The number of defects is very low	1	2	3	4	5
2.c Inventory levels are low	1	2	3	4	5
2.d Productivity has increased over time	1	2	3	4	5

### 3.2. Application of the MCQ

The MCQ is applied to four manufacturing systems in Ciudad Juarez, Chihuahua, Mexico. The surveyed people are employees of middle and senior management with enough knowledge and cultural level to be aware of the macroergonomic practices applied in their companies, their deficiencies and improvement opportunities. There are two steps to follow to apply the survey:

(1) Contacting the company manager by accessing the directories of the National Institute of Statistics, Geography and Informatics (Instituto Nacional de Estadística, Geografía e Informática, INEGI) and the Maquiladoras Association, Civil Association (Asociación de Maquiladoras, Asociación Civil, AMAC). Each manager is contacted by email or telephone; then, he or she is introduced to the project, the objectives and the benefits that may result for the company from its participation in the project. At this step, managers are already familiar with the MCQ.
(2) Setting an appointment to apply the MCQ. The managers involved contacted their colleagues and informed them about the project. Afterwards, managers scheduled an appointment indicating the time, date and place to apply the MCQ.

The MCQ is applied during several days for each of the manufacturing systems according to the availability of the employees and the average time taken to answer the MCQ. All middle and high management staff members are invited to participate in the study. All participants voluntarily agreed to answer the MCQ. In the end, 158 surveys were gathered. Table 6 shows the number of surveyed employees by company.

**Table 6:** Employees surveyed by company.

Company	Company 1	Company 2	Company 3	Company 4
Number of individuals surveyed	41	41	34	42

### 3.3. Statistical Analysis of the Data

Statistical analysis of the data comprises the MCQ statistical validation and the analysis of the relationships among the constructs. This analysis is performed to test the hypotheses proposed in the previous section.

#### 3.3.1. MCQ Statistical Validation

The complete database is made using SPSS^®^ software [80]. A proper data screening is performed by substituting the outliers and missing values by the median, since the data are gathered in an ordinal scale (Likert scale) [78,81,82,83].

Then, the statistical validation of the MCQ is performed for each dimension using Cronbach’s alpha coefficient, considering a minimum cutoff value of 0.7 [78,83,84,85,86,87]. At this step, several variables are removed by considering the corrected total-item correlation and the improved Cronbach’s alpha value obtained after its removal. All variables with a corrected total-item correlation lower than 0.3 are removed only if Cronbach’s alpha coefficient remained above 0.7. The average variance extracted (AVE) is used as an indicator of discriminant validity and convergent validity. For the convergent validity, it is recommended to set a minimum value of 0.5 for each item; meanwhile, the *p*-value has to be significant [78,83,85,87,88].

The index of variance inflation factors (VIFs) is used to detect collinearity between latent variables. Collinearity is accepted as long as the value is below 3.3 in every dimension or latent variable tested [78,89,90]. Moreover, the Q-squared coefficient is used as a nonparametric measure of predictive validity, since data are expressed in an ordinal scale. Validity is achieved when the Q-squared value is higher than zero [78,83,88].

#### 3.3.2. Analysis of the Structural Model

Although there are several techniques to find the relations existing between variables, when the analysis includes several independent and dependent variable, as in this project, the best technique is the use of a structural equation model (SEM) [78].

Once the final variables that formed each construct are defined, the analysis is performed to detect the relations among these constructs to test the hypotheses. All of the hypotheses defined in Figure 1 are tested. The analysis is performed with the aid of the WarpPLS4^®^ software, which does not use a conventional “linear” regression algorithm, but a sophisticated series of algorithms based on partial least squares (PLS) to analyze the data, enabling the use of non-linear models [88,91]. Then, fitting indexes, such as chi-squared, the root mean squared error of approximation (RMSEA) and the goodness of fit index (GFI), are not provided, since they result in being irrelevant [91]. Moreover, WarpPLS4^®^ is widely recommended for small-sized samples [78,88], as is the case in this paper.

Additionally, the model fit and quality indices used, and highly recommended to evaluate the model, are the average path coefficient (APC), average R-squared (ARS) and average variance inflation factor (AVIF) [78,88,92]. For the APC and ARS, the *p*-value is the general criterion to accept or reject a relation between constructs. Because the analysis is performed by using a confidence interval of 95%, relations with *p* < 0.05 are considered significant; otherwise, they are regarded as insignificant and are removed. Once significant relations are detected, the load values are analyzed. If a variable possesses a greater load value in one dimension different from where it belongs, this variable is removed.

The hypotheses to be tested are the null hypothesis (H_a_) that APC and ARS are zero, against the alternative hypothesis (H_b_) that APC and ARS are different from zero. For the AVIF, 5 is set as the maximum acceptable value [78,88].

The direct effect, which indicates a direct relation between dimensions [78], is used to validate the hypotheses depicted in Figure 1, although indirect effects are also measured. Indirect effects are obtained through other dimensions or latent variables, and they can also be due to two or more segments [78]. The sum of direct effects and indirect effects provided a total effect. Hypotheses H_a_ and H_b_ are considered to validate every kind of effect on each parameter:

(1) $$H_{a}:\beta_{1}=0$$

(2) $$H_{b}:\beta_{1}\neq 0$$

## 4. Results and Discussion

This section provides the findings obtained from the models. The section is divided into subsections according to the steps described in Section 3.

### 4.1. Statistical Analysis of the Data

This section provides the results of two different statistical analyses applied to data: statistical validation of the MCQ and analysis of the structural model.

#### 4.1.1. MCQ Statistical Validation

None of the dimensions were removed according to the criteria established in Section 3.3.1. However, some items were removed from different dimensions. For instance, from Table 1, Item 1.3e was removed from the dimension “THAQ conditions,” since it had a corrected total-item correlation below 0.3. It was also removed from the final hypothetical causal model analysis.

After the model was tested in WarpPLS^®^, only Item 1.1c from Table 1 was removed, since it possessed a Cronbach’s alpha value lower than 0.7. Removing this item (which had the lowest value of corrected total-item correlation, weight and loadings) enabled obtaining a Cronbach’s alpha value greater than 0.7. The other final dimensions (lighting, psychological characteristics and employees’ performance) kept their initial number of items (see Table 1).

Table 7 shows the model fit and quality indices. The APC and the ARS are both greater than zero, and the *p*-values are less than 0.001. Hence, the relationships depicted in Figure 3 are significant, and the null hypothesis (*β* = 0) can be rejected. The Tenenhaus goodness of fit (GoF) value is 0.464, meaning that the model as a whole has a large overall explanatory power and predictive quality [88]. Besides, the AVIF and average full collinearity variance inflation factor (AFVIF) values are both below the cutoff value 3.3. The values of Sympson’s paradox ratio (SPS), the R-squared contribution ratio (RSCR) and the statistical suppression ratio (SPR) are equal to one, which means that all of the paths in the model follow the correct direction, and the latent predictive variables have a positive contribution to the R-squared value. Moreover, the model is free of statistical suppression instances. The value of nonlinear bivariate causality direction ratio (NLBCDR) that equals one means that the hypothesized directions of causality are correct.

**Table 7:** Model fit and quality indices.

Index	Value
APC	0.265 *
ARS	0.314 *
AARS	0.303 *
AVIF	1.427
AFVIF	1.615
GoF	0.464
SPR	1.000
RSCR	1.000
SSR	1.000
NLBCDR	1.000

APC, average path coefficient; ARS, average R-squared; AARS, average adjusted R-squared; AVIF, average variance inflation factor; GoF, Tenenhaus goodness of fit; SPR, statistical suppression ratio; RSCR, R-squared contribution ratio. SSR, statistical suppression ratio; NLBCDR, nonlinear bivariate causality direction ratio; * Significant at the 99.9% confidence level.

Values of significance at the 99.9% confidence level mean that although the analyses were performed at a confidence interval of 95%, the results are significant at 99.9%, since the *p*-values are less than 0.001. 

Table 8 presents the validation results for the latent variables analyzed (environmental elements, psychological characteristics and employees’ performance). According to the values obtained, it can be concluded that the MCQ is reliable, since the Cronbach’s alpha value is higher than 0.7 in all of the dimensions analyzed. Furthermore, the AVE values are greater than the minimum cutoff value of 0.5 in all dimensions; thus, the survey has discriminant and convergent validity.

**Table 8:** MCQ validation. AVE, average variance extracted.

Index	*Noise*	*Lighting*	*THAQ Conditions*	*Psychological Characteristics*	*Employees’ Performance*
R-squared				0.281	0.346
Composite reliability	0.892	0.872	0.861	0.912	0.838
Cronbach’s alpha	0.757	0.707	0.782	0.879	0.742
AVE	0.805	0.774	0.614	0.676	0.564
Full VIF	1.464	1.498	2.005	1.603	1.507
Q-squared				0.290	0.354

Table 8 also shows that all R-squared values are acceptable for all latent dependent variables because they are above 0.02. Although there is a lack of agreement about the maximum value of VIF for collinearity, if VIF is less than 3.3, it can be stated that there are no problems of collinearity between latent variables. Therefore, in this research paper, there are no issues of collinearity among latent variables. Finally, every Q-squared value for each dependent variable is higher than zero; hence, nonparametric predictive validation is achieved.

#### 4.1.2. Effects among Variables

As was commented on in Section 3, this study analyzes the direct and indirect effects among latent variables. The sum of direct and indirect effects among latent variables is called the total effects.

##### Direct Effects (Hypotheses Test)

**Figure 2:**
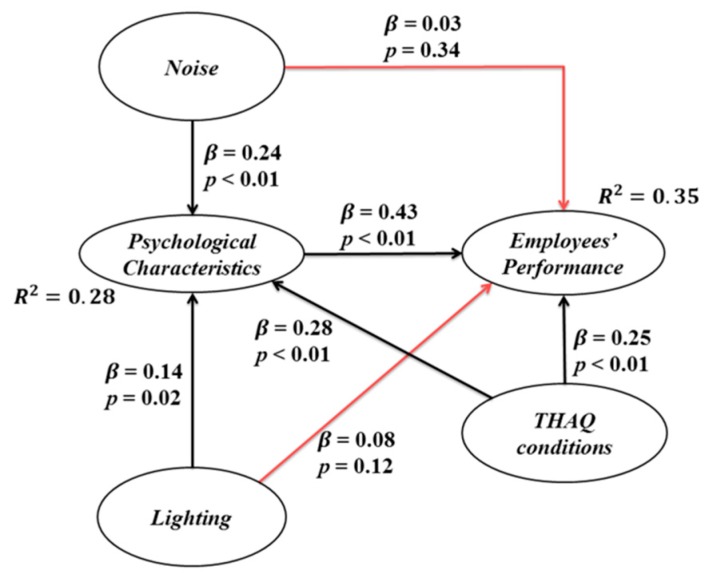
Direct effects.

**Figure 3:**
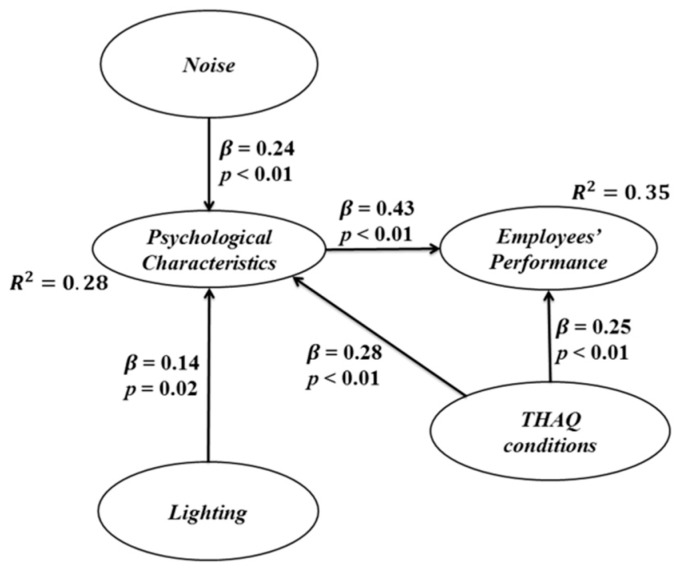
Significant direct effects.

Figure 2 shows all of the direct effects. The values expressed in *β* are dependence measurement values and represent standardized values. *p*-values are the values for the significance hypothesis test. Note that most of the *p*-values are lower than 0.01, which means that the relationships are significant. However, the *p*-values for the relations between noise and employees’ performance and lighting and employees’ performance are both higher than 0.05, meaning that these relations are not significant and can be rejected. It can be seen that THAQ had the highest direct effect on psychological characteristics. Figure 3 only presents the significant direct effects.

Figure 3 also presents the R-squared values, which represent the contribution of independent (exogenous) variables to dependent (endogenous) variables. They also reflect the percentage of explained variance for each of these latent variables [88]. The R-squared value for psychological characteristics is 0.28, which means that this variable is explained at 28% by the independent variables, of which 10.2% is given by the noise, 4.9% by lighting and 13.1% by THAQ conditions. On the other hand, the endogenous variable employees’ performance is explained at 35% by the independent and intermediate variables, of which 23.5% is given by *psychological characteristics* and 11.1% by THAQ conditions.

As far as the *β* values (dependency measures) are concerned, similar interpretations can be provided for each one of them. For instance, the relationship between noise and psychological characteristics has a *β* value of 0.24, which means that when the first dimension increases its standard deviation by one unit, the second dimension increases by 0.24 units. It must be observed that the latent independent variable with the highest *β* values is the variable THAQ conditions; however, as an intermediate latent dependent variable, the psychological characteristics have the highest *β* value. This demonstrates the long-term direct effects that psychological characteristics can have on the manufacturing systems.

According to the values in Figure 3, the structural equations for the dependent latent variables can be stated as:

(3) Psychological Characteristics=0.24×Noise+0.14×Lighting+0.28×THAQ conditions+Error

(4) Employees' Performance=0.43×Psychological Characteristics+0.25×THAQ conditions+Error

##### Indirect Effects

Indirect effects occur between dimensions through other dimensions that act as intermediaries; they can also occur in several segments [78]. Table 9 presents the sum of indirect effects between latent variables analyzed and the corresponding *p*-values. According to the *p*-values, it can be noticed that only the indirect effects of noise and THAQ conditions over employees’ performance are significant, with THAQ conditions having the highest direct effect. Meanwhile, the effect of lighting over employees’ performance is insignificant.

**Table 9:** Sum of indirect effects.

To	From		
	*Noise*	*Lighting*	*THAQ Conditions*
*Employees’ Performance*	0.101	0.058	0.119
*p*-values	0.013	0.099	0.005

All indirect effects can have similar interpretations. For instance, the indirect relation between noise and employees’ performance has an indirect effect of 0.10. This means that when the first dimension increases its deviation by one unit, the second increases by 0.101 units. It must be noticed that THAQ conditions have the most significant indirect effects on the variable *employees’ performance*. This can be considered as a basis for ergonomic work system designs.

##### Total Effects

Total effects are defined as the sum of direct and indirect effects [78]. Table 10 introduces the total effects among the dimensions analyzed. Most impacts are significant at the 99.9% confidence level. However, the majority of the total effects of environmental latent variables (noise, lighting and THAQ conditions) over employees’ performance are significant at least at 98%, with the exception of the effect caused by lighting, which is insignificant. The effects of THAQ conditions on employees’ performance are significant at 99.9%. In general, environmental variables have more significant effects on psychological characteristics, which in turn have the most significant total effect on employees’ performance.

Total effects benefit from the same interpretation as indirect effects. For example, the total effect between noise and employees’ performance is 0.101, meaning that when the first variable increases its standard deviation by one unit, the second increases by 0.101 units, as well.

THAQ conditions have the highest total effect over psychological characteristics, while this variable has the highest total effect over employees’ performance, followed by THAQ conditions.

**Table 10:** Total effects.

To	From			
	*Noise*	*Lighting*	*THAQ Conditions*	*Psychological Characteristics*
*Psychological Characteristics*	0.236 *	0.136 **	0.276 *	
*Employees’ Performance*	0.101 **	0.058 ***	0.365 *	0.430 *

* Significant at the 99.9% confidence level; ** significant at the 98% confidence level; *** insignificant.

## 5. Conclusions and Recommendations

### 5.1. Conclusions Related to the Hypothesis

According to the results shown in Figure 3, Table 9 and Table 10, THAQ conditions are the independent environmental variables that present the highest effects on psychological characteristics and employees’ performance in manufacturing systems. Noise and lighting have no direct effects on employees’ performance, but they do have direct effects on psychological characteristics, which in turn impact employees’ performance.

It can be concluded that environmental variables combine their effects on psychological characteristics. Then, these psychological characteristics cause an impact on employees’ performance. Moreover, THAQ conditions and psychological characteristics seem to have relevant direct effects on employees’ performance in manufacturing systems. This does not mean that manufacturing systems should not pay attention to the elements of noise and lighting in workplaces’ design, since incompatible levels of these elements may have negative effects on psychological characteristics and then on employees’ performance. It rather means that employees in manufacturing systems nowadays tend to feel immune to these environmental variables to perform their tasks.

Furthermore, the results in Figure 3 can be explained as follows:

- Macroergonomic compatibility of noise, lighting and THAQ conditions is required for macroergonomic compatibility of psychological characteristics in manufacturing systems.
- Macroergonomic compatibility of psychological characteristics is required for macroergonomic compatibility of employees’ performance in manufacturing systems.

The following conclusions can be drawn from the hypotheses stated in Section 2:

H_1_: There is enough statistical evidence to state that the macroergonomic compatibility of noise has a direct positive impact on the macroergonomic compatibility of psychological characteristics from employees in manufacturing systems. When the first latent variable increases one standard deviation, the second one increases by 0.24 units.
H_2_: There is not enough statistical evidence to state that the macroergonomic compatibility of noise has a direct positive impact on employees’ performance in manufacturing systems.
H_3_: There is enough statistical evidence to state that the macroergonomic compatibility of lighting has a direct positive impact on psychological characteristics in manufacturing systems, since when the first latent variable or dimension increases its standard deviation by one unit, the second one increases by 0.14 units.
H_4_: There is not enough statistical evidence to state that the macroergonomic compatibility of lighting has a direct positive impact on employees’ performance in manufacturing systems.
H_5_: There is enough statistical evidence to state that the macroergonomic compatibility of THAQ conditions has a direct positive impact on psychological characteristics in employees of manufacturing systems. When the first latent variable or dimension increases its standard deviation by one unit, the second one increases by 0.28 units.
H_6_: There is enough statistical evidence to state that the macroergonomic compatibility of THAQ conditions has a direct positive impact on employees’ performance in manufacturing systems, because when the first latent variable or dimension increases its standard deviation by one unit, the second one increases by 0.25 units.
H_7_: There is enough statistical evidence to state that the macroergonomic compatibility of psychological characteristics has a direct positive impact on employees’ performance in employees of manufacturing systems, because when the first latent variable or dimension increases its standard deviation by one unit, the second one increases by 0.43 units.

Hypotheses H_2_ and H_4_ were not statistically significant since their *p*-values were greater than 0.05, which means that there is not enough statistical evidence to state that noise and lighting have an effect on employees’ performance on the sample analyzed. This may be because variation of noise and lighting is low in the workplaces analyzed.

The methodology presented here is original, since it relates macroergonomic compatibility of environmental elements to employees’ psychological characteristics and performance. With respect to the MCQ, it is concluded that this questionnaire is a new and effective instrument to collect information about macroergonomic practices and the frequency with which they are applied in manufacturing systems, which in turn can help measure the macroergonomic compatibility of these systems by means of statistical or mathematical methods.

### 5.2. Industrial Implications

On the one hand, it is recommended that manufacturing systems, and companies in general, design a work system by considering macroergonomic factors, because they can provide long-term benefits. Furthermore, this paper recommends ensuring employees’ satisfaction by taking into account their needs. This can become a competitive advantage for the company, since satisfaction means motivation. Finally, the paper also recommends companies be open to changes in their work system’s design and to apply ergonomics practices at all levels of their work systems (micro- and macroergonomics).

### 5.3. Future Research

The authors recommend expanding macroergonomic research to other fields, such as education, construction, health and commerce, more specifically in supermarkets, not only to determine if macroergonomic practices are applied in these fields, but also to apply them and perform a before and after comparative analysis of the outcomes that companies may obtain. The paper also recommends continuing searching for a universal matrix to measure ergonomic compatibility.
